# Fixation of the Esophagus to Diaphragmatic Hiatus as a Routine Step in Hiatal Hernia Repair During Bariatric Surgery: How to do it? A Multimedia Article

**DOI:** 10.1007/s11695-025-07804-w

**Published:** 2025-04-14

**Authors:** Emad Abdallah, Ibrahim Saleh Alawadi

**Affiliations:** 1 Professor of general surgery, Department of General Surgery, Mansoura University Hospital, 60 Gomhouria street, Mansoura, 35516 Egypt; 2 Mansoura Manchester Program for Medical Education, Faculty of Medicine, Mansoura University Hospitals, Mansoura, Egypt

**Keywords:** Laparoscopic gastric sleeve, Hiatus hernia, Hiatal hernia repair, Esophagus fixation, Intrathoracic migration, “De novo”, Phreno-esophageal membrane, Bariatric surgery, Video

## Abstract

**Introduction:**

This approach aims to reduce postoperative intrathoracic migration (ITM) of the esophagus and upper gastric tube, decreasing the recurrence of hiatal hernia (HH) and the development of “de novo” HH after Hiatal hernia repair (HHR) if indicated in bariatric surgery, especially laparoscopic sleeve gastrectomy (LSG).

**Methods:**

In performing LSG, HHR is conducted if indicated based on involving both pre-operative and intra-operative evaluations. Following this, our innovative technique is applied, which involves esophageal fixation to the diaphragmatic hiatus after a complete dissection of the phreno-esophageal membrane to free the full length of the intra-abdominal esophagus. This is accomplished through two distinct methodologies: interrupted suture fixation and continuous suture fixation using a 2/0 Ti-Cron^TM^ suture over a 26-mm round needle, both commonly used in HHR. These techniques are comprehensively described in the accompanying video.

**Results:**

All patients are discharged from the hospital the next day and followed up at the clinic after the end of the first and second weeks, then after 3, 6, and 12 months postoperatively.

**Conclusion:**

Combining esophageal fixation with HHR during LSG is assumed to reduce ITM and the possibility of HH recurrence, improving patient quality of life.

**Supplementary Information:**

The online version contains supplementary material available at 10.1007/s11695-025-07804-w.

## Introduction

Over a billion people worldwide are suffering from obesity. Since 1990, obesity has more than doubled globally in adults, as in 2022, 43% of adults were overweight [1]. This is serious as obesity increases overall mortality by 29% for every 5-unit increase in Body Mass Index BMI over 25 kg/m^2^ [2]. Bariatric surgeons are increasingly recognizing laparoscopic sleeve gastrectomy (LSG) as an effective treatment for obesity. LSG has low morbidity and mortality rates and reduces obesity-related comorbidities [3, 4]. Upper gastrointestinal investigations reported that almost 40% of severely obese patients had a hiatal hernia (HH), and surgical hiatal hernia repair (HHR) by the time of LSG is recommended [5, 6].

There is a growing concern about post-LSG-induced gastroesophageal reflux disease (GERD) due to either “de novo” HH or its recurrence following HHR because of postoperative intrathoracic migration (ITM) of the esophagus and the upper gastric tube. Rapid excess weight loss (EWL) after LSG may increase the risk of HH recurrence due to enlarged natural orifices, muscle depletion causing hypotonic diaphragm pillars, and dissection of the angle of His and left pillar during gastric tube creation, which may increase herniation risk [7]. It results in impairment in patients' quality of life, besides a possible risk of further evolution to Barrett’s esophagus or even to esophageal adenocarcinoma itself [8].

This technique aims to prevent ITM, HH recurrence, and “de novo” HH after bariatric surgery, especially LSG, by esophageal fixation as a routine step in HHR for indicated patients besides bariatric surgery.

## Methods

Our practice at our center (a high-volume bariatric and metabolic surgery) has developed a more nuanced approach over the past 2 years. The decision to perform HHR with LSG is based on a cautious evaluation involving both pre-operative and intra-operative considerations. Pre-operatively, triple assessment is performed: a comprehensive questionnaire into clinical reflux symptoms, diagnostic pre-operative oesophago-gastroduodenoscopy (OGD) screening based on Hill’s classification [9] for categorization from stages one to four, and, only when indicated, a virtual computed tomography (CT) scan. In the intra-operative assessment, the presence of HH or a notably lax hiatus is confirmed [10].

By the time of performing LSG with T-shaped omentoplasty [11], HHR is performed as standard practice. Subsequently, our novel technique is implemented, involving esophageal fixation repair to the diaphragmatic hiatus utilizing two distinct methodologies: interrupted suture fixation and continuous suture fixation as a routine step in HHR, which is meticulously detailed in the provided video, Informed consent was obtained from the patients included in this video.

### Fixation of the Esophagus

We begin with the dissection at the left crus, exposing the white line, which is the area between the phreno-esophageal membrane and the left crus of the diaphragm; routinely excise the sub hiatus (esophageal) fat pad; continue the dissection through the right crus; upon reaching the appropriate plane, the lateral wall of the esophagus will become visible; proceed posteriorly to expose both the left and right crura; perform an anterior dissection to fully dissect the remaining portion of the phreno-esophageal membrane, ensuring the full length of the intra-abdominal esophagus is obtained. Be cautious to avoid injuring the esophageal wall, anterior and posterior vagus nerves, aorta, pleura, and pericardium by employing traction and counter-traction techniques. Approximate crura using 2/0 Ti-Cron^TM^ suture over a 26-mm round needle. Subsequently, insert a 38-French gastric calibration tube (38-Fr bougi) to confirm the absence of esophageal kinking. As an interrupted suture fixation method, the first suture is placed between the anterior esophageal musculature and the hiatus. The second suture is placed between the esophageal wall and the right crus to approximate the esophagus laterally. Finally, the third and final sutures are placed between the esophageal wall and the left crus. Furthermore, according to continuous suture fixation, we utilize a 2/0 Ti-Cron^TM^ suture over a 26-mm round needle to fix the esophageal muscular wall and the diaphragmatic crura continuously, as presented in the video. Typically, we encircle the anterior half of the esophageal wall.

## Results

According to the interrupted suture fixation method, usually, about 10 min and about 5 min in the continuous suture fixation method are added to the total operative timing. The esophagus fixation added no extra days in the hospital without any surgical drain or nasogastric tube. We developed this standardized technique in more than 2 years regarding the HHR side by LSG. Follow-up of all patients is done in the outpatient clinic after the end of the first and second weeks, then after 3 months, 6 months, and 1 year postoperatively. This is a technical note describing the methodology of the technique. Data collection and analysis are ongoing and have not yet been completed or published.

### Limitations

This study has the following limitations: (1) single-center design. Future multicenter studies with larger sample sizes and longer follow-ups are necessary to validate the results. (2) Multimedia focus: This is a technical note presented as a multimedia article. The clinical outcomes are still under investigation, and the data has not yet been published. (3) Pre- and postoperative assessments using standardized tools are needed to accurately determine the efficacy and safety of this technique.

## Conclusion

Fixation of the esophagus to the diaphragmatic hiatus as a routine step in HHR during LSG may help reduce the risk of HH recurrence and the development of “de novo” HH post-LSG. By minimizing ITM of the esophagus and the upper gastric tube, this technique has the potential to enhance patients’ QoL and satisfaction levels. However, proper objective data collection and analysis are essential to confirm the efficacy of this technique. Long-term follow-up studies are also necessary to validate these findings.

## Supplementary Information

Below is the link to the electronic supplementary material.

Supplementary file1 (MOV 141960 KB)

## Data Availability

No datasets were generated or analysed during the current study.

## Abbreviations

BMI: Body mass index
CT: Computed tomography
T2DM: Type 2 diabetes mellitus
EWL: Excess weight loss
GERD: Gastroesophageal reflux disease
HH: Hiatal hernia
ITM: Intrathoracic gastric migration
HHR: Hiatal hernia repair
LSG: Laparoscopic sleeve gastrectomy
OGD: Esophago-gastro-duodenoscopy

## Declarations

Ethical Approval

This video presentation does not require ethical approval.
